# Ingested asbestos in filtered beer, in addition to occupational exposure, as a causative factor in oesophageal adenocarcinoma

**DOI:** 10.1038/s41416-019-0467-9

**Published:** 2019-05-09

**Authors:** Rebecca C. Fitzgerald, Jonathan M. Rhodes

**Affiliations:** 1 MRC Cancer Unit,Hutchison-MRC Research Centre, University of Cambridge, Hills Road, Cambridge, CB2 0XZ USA; 2 Department of Cellular and Molecular Physiology, Institute of Translational Medicine, University of Liverpool, The Henry Wellcome Laboratory, Nuffield Building, Crown St., Liverpool, L69 3GE UK

**Keywords:** Oesophageal cancer, Oncogenesis

## Abstract

Oesophageal adenocarcinoma has become much more common over the past 50 years, particularly in Britain, with an unexplained male to female ratio of > 4:1. Given the use of asbestos filtration in commercial brewing and reports of its unregulated use in British public houses in the 1970’s to clear draught beer “slops”, we have assessed the hypothesis that ingested asbestos could be a causative factor for this increased incidence. Importantly, occupational asbestos exposure increases the risk of adenocarcinoma but not squamous cell carcinoma of the oesophagus. The presence of asbestos fibres was consistently reported in filtered beverages including beers in the 1970s and asbestos bodies have been found in gastrointestinal tissue, particularly oesophageal tissue, at autopsy. There is no reported association between the intake of alcohol and oesophageal adenocarcinoma but studies would mostly have missed exposure from draught beer before 1980. Oesophageal adenocarcinoma has some molecular similarities to pleural mesothelioma, a condition that is largely due to inhalation of asbestos fibres, including predominant loss of tumour suppressor genes rather than an increase of classical oncogenic drivers. Trends in incidence of oesophageal adenocarcinoma and mesothelioma are similar, rising rapidly over the past 50 years but now plateauing. Asbestos ingestion, either from beer consumed before around 1980, or from occupational exposure, seems a plausible causative factor for oesophageal adenocarcinoma. If this is indeed the case, its incidence should fall back to a low baseline by around 2050.

## Background

There has been a dramatic increase, approximately six-fold, in the incidence of oesophageal adenocarcinoma over the past 50 years,^1^ but with a very uneven geographical distribution and a striking male predominance. Oesophageal adenocarcinoma has become much more common in northern and western Europe, North America, and Oceania than in other parts of the world.^2^ Large differences also exist between western European countries, with incidence rates (per 100,000 men) in the UK (6.33 in 2005) and the Netherlands (5.32) that far exceed those in other European countries including Denmark (2.80), France (1.87), Spain (1.29) Slovakia (0.81), Italy (0.76), and Croatia (0.60).^3^ The very marked male predominance−up to 9:1 male-to-female ratio−is also unexplained.^4^ Increasing rates of obesity and the decline in *Helicobacter pylori* gastritis, with its possible consequential increase in acidity of refluxate, can only offer partial explanations for the increased incidence. The pre-malignant condition, Barrett’s oesophagus, in which intestinal metaplasia affects a variable length of the lower oesophagus, changing it from a squamous to a columnar epithelium, is strongly associated with gastro-oesophageal reflux. Reflux in turn is strongly associated with risk for oesophageal adenocarcinoma but modelling has estimated that other factors account for 78% of the increasing incidence in men and 33% of the increase in women.^5^ Barrett’s oesophagus also shows a male predominance, but this is much less marked with an overall risk ratio of just under 2:1.^4^

The increase has not impacted equally on all ages, suggesting a possible cohort effect.^6^ Thus, in England where the incidence of adenocarcinoma rose more than three-fold between 1972 and 2012, incidence rates per 100,000 for men aged 40–49 years only increased from 1.7 to 3.1 whereas rates for those 80 years and above increased from 23.0 to 84.1.^7^ Moreover, the overall incidence of oesophageal adenocarcinoma has been levelling off since ~2010 ^8^ and possibly even starting to fall in countries with a high incidence such as England^7^ and Denmark.^3^ In this article we review the possibility that ingestion of asbestos, used as a filtering agent in alcoholic drinks, particularly beers, up to the late 1970s might be a causative factor for oesophageal adenocarcinoma.

## Asbestos filtering of beers and other alcoholic drinks

Asbestos filtering has in the past been used routinely in the alcoholic drinks industry to clear sediments and microorganisms from beer and other drinks prior to bottling.^9^ This procedure has a long history - thus, from 1914: “To be successful in chilled beer bottling the filters and the pulp are of the first importance…. best beer asbestos should be added once a week at the rate of 8oz. per cwt. of pulp being washed …care must be taken not to add too much asbestos, otherwise clogging of the filter plates ensues. The asbestos should always be whisked up to a cream with water and added slowly to the circulating pulp.^10^” While we cannot be sure which type of asbestos was used for beer filtration, the widespread use of chrysotile in the wine filtration industry makes this the most likely candidate for beer filtration (Box 1). Furthermore, an electron microscopy study of ashed centrifugates from six samples of commercially bottled and canned beers, published in 1968, revealed what were thought to be chrysotile asbestos fibres at an average of around 5000 fibres per pint.^11^ A subsequent study showed even higher concentrations of asbestos fibre in beer but with still higher concentrations in some samples of filtered public tap water supplies in Canadian cities.^12^ Various anecdotal sources suggest that asbestos filtering during commercial manufacture of alcoholic drinks probably continued into the late 1970s.^13^

In 1973, when one of the authors (JMR) was a house surgeon in London, a middle-aged male patient was admitted to have a fingertip amputated for a Bowen’s tumour (squamous cell carcinoma-in-situ) of the nail-bed. The patient acknowledged that he was exposed to asbestos in his occupation as a publican (landlord/manager of a British public house (“pub”) or bar). He explained that it was common practice to take the “slops” (the beer that had splashed into a bucket below the hand pump used for serving draught beer in a typical British pub) at the end of the day, add a slurry of asbestos and then run them through a filter – he made a circular swishing gesture with his hand to show how this was done. The asbestos-filtered “slops” were then served to the (presumably unsuspecting) first customers into the pub the next day. The patient thought this practice was quite common at that time. It seems likely that this filtration technique, if widely used, would have given even greater exposure to people, principally men, drinking draught beer in British pubs during the time, perhaps decades, when this practice was used. Thus, although commercial use of asbestos filtering in alcoholic drinks was a common practice across many countries, the much cruder use of asbestos filtration in public houses might have given particularly high exposure to British beer drinkers.

Box 1 Various asbestos fibres and their use in filters Asbestos fibres and filters

Asbestos comprises a group of six naturally occurring silicate minerals typified by long fibrous crystals. All are regarded as carcinogenic. As the sole, curly fibred, member of the serpentine group of asbestos, chrysotile (white asbestos) accounts for about 90–95% of worldwide use, and probably confers a substantially lower mesothelioma risk than members of the amphibole group which are straight and needle-like and include amosite (brown asbestos) and crocidolite (blue asbestos), as well as tremolite, actinolite and anthophyllite.^60^ The use of chrysotile for wine filtration has been clearly documented^61^ and this seems the most likely fibre type for use in beer filtration^12^ although the more dangerous crocidolite was notoriously used in the manufacture of cigarette filters.^62^

## Ingested asbestos as a risk factor for oesophageal adenocarcinoma

Epidemiology studies have failed to show any convincing association between alcohol consumption and the risk of oesophageal adenocarcinoma;^8^ however, any association with exposure to asbestos in alcoholic drinks, particularly beer, is likely to have been missed. This is partly because only those people who were adults in the 1970s or earlier would have been exposed, and alcohol consumption during that time period would not have been assessed in the association studies, which were not performed until at least ten years later and usually addressed current or recent drinking habits which are less relevant given the lengthy lag period between asbestos exposure and cancer risk. Moreover, asbestos exposure might be very variable depending on the type and source of the alcohol consumed. For example, the relatively low, but rising, risk for oesophageal adenocarcinoma amongst African-American males^14^ might relate to a higher proportion of alcohol intake from spirits rather than beers, particularly in the 1970s when risk of asbestos contamination of beer is likely to have been greatest.^15^

A 2016 meta-analysis of 20 cohort studies has shown that occupational exposure to asbestos is associated with a moderately increased risk of oesophageal cancer (standardised mortality ratio (SMR) 1.24; 95% CI 1.13,1.38, *P* < 0.001) but did not differentiate between adenocarcinoma and squamous cell carcinoma.^16^ In the prospective Netherlands Cohort Study of 58,279 men followed over a mean of 17.3 years, a significant association between occupational asbestos exposure was found for adenocarcinoma of the oesophagus but not for squamous cell carcinoma.^17^ For ever versus never highly exposed subjects, the hazard ratio (HR) for adenocarcinoma of oesophagus was 2.52 (95%CI 1.01,6.26) with a significant exposure-response relationship both for duration of exposure and cumulative exposure (*P* < 0.05). These results support an earlier study from Sweden showing an incidence rate ratio of 4.5 [95%CI 1.4,14.3] for occupational asbestos exposure and adenocarcinoma of the oesophagus but, again, no significant association with squamous cell carcinoma.^18^

It is presumed that asbestos fibres are ingested either by contamination of food/drink or by swallowing sputum following inhalation. It should be noted that the strength of association between occupational asbestos exposure and oesophageal adenocarcinoma is relatively modest compared with that for occupational exposure and pleural mesothelioma in which odds ratios might be as high as 50;^19^ it is plausible, though, that asbestos in filtered beverages, particularly beer, might have accounted for the major source of ingested asbestos and would have been missed by studies of oesophageal adenocarcinoma based on occupational asbestos exposure. The latter studies typically assess exposure to building materials such as asbestos insulating boards, lagging or cement, or a surrogate for this such as construction or shipyard working or carpentry. One study has investigated the association between exposure to asbestos and the risk of Barrett’s oesophagus, finding a possible trend that was not significant on multivariate analysis.^20^

### Asbestos as a risk factor for adenocarcinoma but not squamous cell carcinoma

It is not surprising that the oesophagus might be particularly susceptible to the harmful effects of ingested asbestos; however, it is less clear why ingested asbestos should be a risk factor for adenocarcinoma but not for squamous cell cancer. A similar increased association between asbestos exposure and adenocarcinoma of the lung (RR 3.31) compared with squamous cell lung cancer (RR1.67) has also been reported.^21^ Possibly the fine asbestos fibres are more likely to penetrate columnar epithelium than squamous epithelium. This would then also help explain why Barrett’s oesophagus, with its columnar epithelium, is such a strong risk factor for oesophageal adenocarcinoma.^22^

One might therefore also expect ingested asbestos to be a risk for gastric and other intestinal cancers. The prospective Netherlands Cohort study did indeed show associations between prolonged exposure to high levels of asbestos and increased risks for gastric cancer (including non-cardia gastric cancer (cancer of the lower stomach)) as well as for colon and rectal cancer.^20^ Mortality rates for both gastric cancer and colorectal cancer in Britain have, however, been steadily declining since the 1970s^23^ indicating that factors other than asbestos, including the falling incidence of *H. pylori* and the impact of bowel cancer screening, are likely quantitatively more important for cancer at those sites.

### Evidence for asbestos in the gastrointestinal tract

Concerns started to be raised in the 1970s about the possible health effects of asbestos in beverages but relatively few studies examined human intestinal samples directly for presence of asbestos.^24,25^ The detection of asbestos fibres ideally requires transmission electron microscopy of concentrated residues after digestion and ashing of tissue samples although for tissue samples (rather than lavage fluid, for example) the identification of asbestos bodies in tissue sections using light microscopy might have equivalent sensitivity.^26^ Autopsy studies of 26 male subjects over 40 years old showed that individuals with the highest concentration of pulmonary asbestos bodies also had asbestos bodies at varying concentrations particularly in the oesophagus (mean 15.5 per gram) and to a lesser extent in the stomach (mean 1.6 per gram), small intestine (mean 1.2 per gram) and colon (1.1 per gram) as well as in other organs, particularly the spleen (mean 6.6 per gram).^27^ A study of patients with colorectal cancer found asbestos fibre and/or asbestos bodies at a mean concentration of approximately 2.5 × 10^6^ per gram in colon tissue from 14 out of 44 patients with a history of occupational asbestos exposure compared with 0 of 20 colon cancer patients without a history of asbestos exposure.^28^ No equivalent systematic analysis of asbestos in oesophageal cancer tissue has been reported, although in two cases–one adenocarcinoma and one squamous cell carcinoma–asbestos bodies were found within tumour tissue in patients with a history of occupational asbestos exposure.^29^ (Supplementary Fig. 1)

## Mechanisms of asbestos-induced neoplasia: comparisons with mesothelioma

The strongest tumour association with asbestos exposure is for mesothelioma, particularly of the pleura. It is therefore worth considering the mechanisms for asbestos carcinogenicity and then to compare the biology of oesophageal adenocarcinoma and pleural mesothelioma.

### Asbestos as a tumour promoter rather than an initiator

Although early in vitro assays suggested that asbestos was not genotoxic, subsequent studies by various investigators demonstrated genotoxic effects including chromosomal aberrations such as aneuploidy.^30–32^ However, the very long incubation period from initial asbestos exposure to tumour manifestation, typically several decades, has been taken to suggest that asbestos is acting as a tumour promoter rather than as an initiator.^30^ Studies on lung cancer tissue have shown that adenocarcinomas tend to exhibit less allelic loss than squamous cell carcinomas and that asbestos exposure correlates with a greater frequency of p53 mutation, which again implies a tumour promoting effect through loss of suppression.^33^

In mesotheliomas, increased mutation frequency has consistently been reported in tumour suppressors such as neurofibromatosis type 2 (NF2)^34,35^ and the nuclear deubiquitinase Bap1.^36^ Monosomy of chromosome 22 or deletions in 22q that include NF2 as well as homozygous deletions in 9p21, which encompass the cyclin-dependent kinase inhibitor 2A (*CDKN2A* locus), are reported in up to 72% cases of mesothelioma,^36^ but there are also frequent deletions at other sites, including 1p, 3p, 4p, 6q, 13q, 14q, and 15q.^37^ Indeed, mesothelioma is considered to be a ‘disease of gene loss’ rather than being associated with classical driver mutations in oncogenes,^38^ which is similar to the situation that has long been appreciated for oesophageal adenocarcinoma.^39^ It should be noted, however, that many of these molecular changes also occur in epithelial cancers that do not have asbestos as a known major factor. The inflammatory response to asbestos fibres, including macrophage activation and associated cytokine release, is also thought likely to be important in tumour promotion.^40^ Notably, gene copy number changes are very common in oesophageal adenocarcinoma with a large number of chromosomal rearrangements reported.^41–44^ It is perhaps not surprising that there is some overlap with the typical chromosomal alterations found in mesothelioma such as 9p21 deletions, which have long been known to occur early in disease pathogenesis, including in premalignant Barrett’s oesophagus.^45–47^ However, in view of the heterogeneity of oesophageal adenocarcinoma^48^ and the limited data sets available for mesothelioma, it is difficult to make precise comparisons with the genetic landscape of mesothelioma.

### Mutational signatures

A complementary approach that has been facilitated by the advent of whole genome sequencing is to identify and classify mutational processes through the statistical analysis of the frequency of base changes (A > C, T > G, etc.) throughout the entire genome, which can also be viewed in the context of the base either side (so-called ‘trinucleotide context’). Through the analysis of many normal and cancer genomes, a number of patterns or so-called ‘mutational signatures’ have been described which can, in some cases, be ascribed to particular mutagens such as UV radiation, cigarette smoke or physiological ageing and a catalogue of these signatures has been compiled using a non-negative matrix factorisation algorithm.^49–51^

The most frequent and specific signatures observed in oesophageal adenocarcinoma are T>G substitutions in a CTT context, called the ‘S17 signature’.^41^ Other signatures reflect ageing (S1); a complex pattern caused by defects in the BRCA1/2-led homologous recombination pathway (S3); C>T mutations in a TCA/TCT context, which is due to mutations in the apolipoprotein B mRNA editing enzyme, catalytic polypeptide-like (APOBEC) proteins (S2); and C>A/T dominant in a GCA/TCT context (S18), which is also found in gastric carcinoma as well as neuroblastoma. The APOBEC signature is also associated with characteristic clusters of localised hypermutation, named kataegis, in which a single strand accumulates a high burden of C>T and C>G mutations.^43^

A similarly rigorous genome-wide mutational analysis has yet to be performed for pleural mesothelioma, although there are data from peritoneal mesothelioma which has a weaker association with asbestos exposure.^52^ These data include signature 5, a transcriptional bias for T>C in an ApTpN context. Although this signature is also found in oesophageal adenocarcinoma, it is present in other cancer types, too, and its aetiology is unknown. To identify the unknown cancer-causing factors, a study to sequence the genomes of 5000 cancers, including oesophageal cancer, from across five continents is currently underway.^53^ Additional genome-wide sequencing data in mesothelioma are required to allow a comparative analysis between pleural mesothelioma and oesophageal adenocarcinoma. (Box 2)

Box 2 Comparison of biology between pleural mesotheliomas and oesophageal adenocarcinoma – it should be noted that many of these changes are common in other tumours also – better evidence for similarities in underlying aetiology might be uncovered when patterns of nucleotide substitutions are known for mesothelioma

**Table 1:** 

Pleural mesothelioma	Oesophageal adenocarcinoma
Increased mutation and deletion frequency in tumour suppressors	Alterations in tumour suppressors including *TP*53 are early events
DNA hypermethylation (including hypermethylation of tumour suppressors) common	Altered DNA methylation (majority with hypermethylation)
Chromosomal alterations include 9p21 deletions	9p21 deletions occur early eg in Barrett’s oesophagus
Pattern of nucleotide substitutions (signatures) not yet known	Signature nucleotide substitutions include T > G in CTT, C > T in TCI/TCT, C > A/T in GCA/TCT with characteristic clusters of localised hypermutation (kataegis)

## Mesothelioma predictions as a possible model for the future of oesophageal adenocarcinoma

**Fig. 1:**
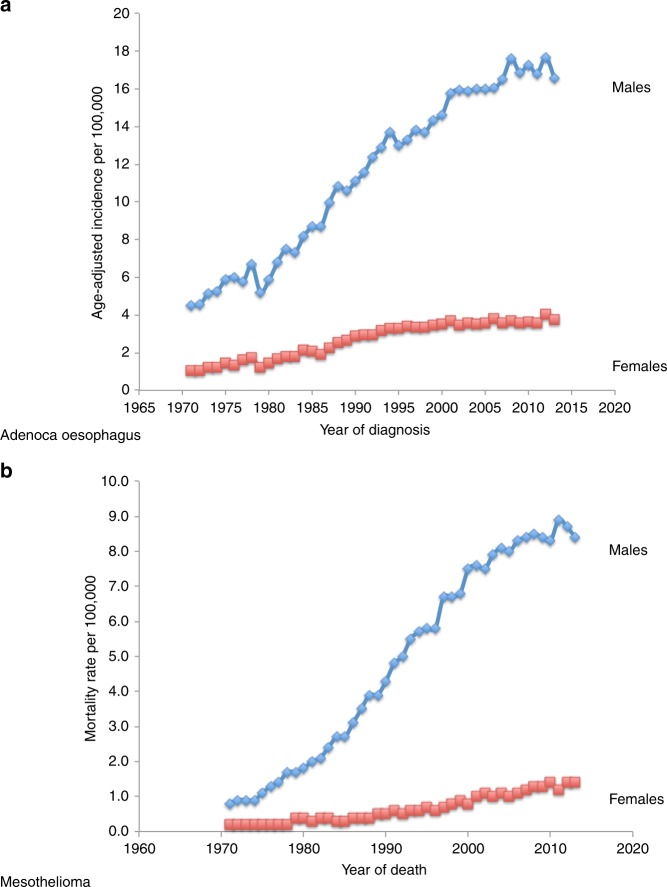
Incidence trends for mesothelioma and oesophageal adenocarcinoma. **a** Age-adjusted incidence of oesophageal adenocarcinoma in England by year of diagnosis (Modified from^7^: *British Journal of Cancer* **118**, 1391–1398 (2018)). **b** European age-standardised mortality rates for mesothelioma in Great Britain by year (Data from Cancer Research UK Cancer Statistics^59^). Note that mesothelioma data are for mortality, but adenocarcinoma oesophagus data are for incidence; however, both conditions have an average life expectancy of around one year from diagnosis, so the trends are approximately comparable

Britain has the highest rate of mesothelioma worldwide but almost all cases relate to exposure before 1980 when asbestos was widely used. Crocidolite use ended in 1970 and amosite use by the late 1970s.^54^ Thus, of 2542 deaths from mesothelioma in 2015, only three of the deceased were born after 1975,^55^ and it has been predicted that asbestos-related mesothelioma should have disappeared in Britain by 2055, by which time anyone born before 1965 will be over 90.^54^ Given that asbestos ingestion from beer should only affect people who had already reached adulthood by the late 1970s, then the epidemic of oesophageal adenocarcinoma, if related to asbestos ingestion from beer rather than occupational exposure, might resolve slightly earlier, perhaps by 2050, and should be reducing well before then. This scenario is substantially more optimistic than has been predicted by statistical analysis using age-period-cohort models,^7^ and is compatible with the similar incidence trends seen for mesothelioma and oesophageal adenocarcinoma (Fig. 1).

Beer will not, of course, be the only source of ingested asbestos; it might plausibly represent the major source for some individuals but could be a relatively less important source than occupational asbestos exposure for other individuals. In relation to this, it is notable that Britain sadly leads the world for mesothelioma mortality rate, as well as for oesophageal adenocarcinoma, and that mesothelioma also has a similar, very strong male predominance – ~4.2 to 1 in Britain,^54^ similar to the 4.5 to 1 ratio for oesophageal adenocarcinoma in England.^7^ A broader hypothesis would therefore be that the epidemic of oesophageal adenocarcinoma might be substantially related to ingested asbestos from all sources including both filtered beers and occupational exposure. A recent study has shown that manufactured carbon nanotubes, which are similar in shape to asbestos fibres, and are widely used in sports equipment, computers and building, might also induce mesothelioma with similar latencies and molecular pathways to asbestos, including early loss of *p16/lnk4a*, suggesting that other modern manufacturing materials should also be examined for their potential carcinogenicity.^56^

## Conclusions and further studies

It seems plausible that ingested asbestos fibres – particularly, but not solely, from beer consumed before 1980 - could be a significant factor in the epidemic of oesophageal adenocarcinoma that has particularly affected males in selected countries. The key points supporting this association are summarised in Box 3. If this hypothesis is correct, then there should be a substantial fall in incidence, hopefully culminating in a return to a low background level by ~ 2050 in the UK. Other countries may have differing incidence trajectories depending on when - and whether – the use of asbestos filtration in brewing has been abandoned.^57^ Indeed, different countries have different time trends for incidence of oesophageal carcinoma^58^ but it is hard to know whether or not these differing time trends relate to differing asbestos exposure. Useful circumstantial supportive evidence might be obtainable from further comparative studies of the molecular alterations in oesophageal adenocarcinoma and pleural mesothelioma, and by further analysis of oesophageal tumour tissue for evidence of asbestos.

Box 3 Key Points: Asbestos ingestion as an explanation for the epidemic of oesophageal adenocarcinoma •Widespread usage of asbestos filtering in brewing and manufacture of alcoholic drinks prior to 1980.

•Uncontrolled usage of asbestos filtering of draught beer in British public houses.

•Association between occupational asbestos exposure and oesophageal adenocarcinoma (not squamous).

•Strong male predominance (c4.5:1M:F) of oesophageal adenocarcinoma.

•Asbestos bodies found in oesophageal tissue at autopsy.

•Similarities in molecular features of oesophageal adenocarcinoma and pleural mesothelioma.

•Similar time trends of oesophageal adenocarcinoma and pleural mesothelioma.

## Supplementary information

Supplementary Figure

## Data Availability

Not applicable (review article – all data already in public domain).
